# Lycium Barbarum Polysaccharides and Wolfberry Juice Prevent DEHP-Induced Hepatotoxicity via PXR-Regulated Detoxification Pathway

**DOI:** 10.3390/molecules26040859

**Published:** 2021-02-06

**Authors:** Huan Liu, Xiong Zhou, Shaowen Huang, Jie Yang, Ruijing Liu, Chunhong Liu

**Affiliations:** Guangdong Provincial Key Laboratory of Food Quality and Safety, College of Food Science, South China Agricultural University, Guangzhou 510642, China; m13710224344@163.com (H.L.); zhoucoon@163.com (X.Z.); seven060721@foxmail.com (S.H.); Judy_yang91@163.com (J.Y.); liuruijing1000@126.com (R.L.)

**Keywords:** *Lycium barbarum* polysaccharides, wolfberry juice, di(2-Ethylhexyl) phthalate, pregnane X receptor, intervention

## Abstract

Environmental di(2-Ethylhexyl) phthalate (DEHP) is widely used in various industries as a plasticizer, and has been reported to induce reproductive and developmental toxicities in organisms. The purpose of this study was to evaluate the detoxification capacity of *Lycium barbarum* polysaccharides (LBP) and wolfberry juice (WJ) against DEHP-induced hepatotoxicity. Two groups of rats were purchased to study two different intervention method experiments: LBP (50, 100, 200 mg/kg·bw) intervention before DEHP (2000 mg/kg·bw) exposure, and LBP (200 mg/kg·bw) or WJ (8 mL/kg·bw) intervention after DEHP (3000 mg/kg·bw) exposure. The rats were exposed to DEHP once, while the intervention lasted for seven days. At the end of the intervention, enzyme-linked immunosorbent assay (ELISA) was used to measure the related index. The LBP intervention before DEHP exposure experiment (the first experimental method) found that LBP group rats showed a strong capacity toward DEHP detoxification, evidenced by the significant upregulation of activities and concentrations of the partner retinoid, X receptor alpha (RXRα), and downstream regulators Cytochrome P4502E1 (CYP2E1), Cytochrome P4503A1 (CYP3A1), Glutathione S-Transferase Pi (GSTpi), and UDP-glucuronosyltransferase 1 (UGT1) in a dose-dependent manner. The LBP and WJ intervention after DEHP exposure experiment (the second intervention experiment) found that WJ could downregulate pregnane X receptor (PXR), and upregulate downstream regulators, CYP2E1, CYP3A1, and Glutathione S-Transferase (GST) with the extension of intervention time, to alleviate the toxicity of DEHP. However, the intervention effect of WJ was more obvious than that of LBP. These results suggested that LBP and WJ might be effective detoxification agents against DEHP-induced toxic effects, by activating PXR and PXR-related detoxifying enzymes.

## 1. Introduction

Di(2-Ethylhexyl) phthalate (DEHP) is the most commonly used phthalate worldwide [1]. From Europe to Asia, DEHP is omnipresent and is commonly found in medical devices, household products, plastics, pharmaceutical preparations, food packaging, and industrial products. Since DEHP is toxic and ubiquitous in water, food, and air, it can be seen that it is very harmful to wildlife and human beings [2]. Di(2-Ethylhexyl) phthalate can disrupt endocrine function [3,4], and exposure to DEHP during the prenatal phase, infant phase, childhood, preadolescent phase, and post-adolescent phase causes severe harm to the body [5,6,7,8]. At the same time, DEHP, as an environmental endocrine disruptor, also has embryotoxicity, immunotoxicity, genotoxicity, and neurotoxicity. Previous results showed that DEHP could damage the reproductive organs, blood, kidney, lung, and liver of experimental animals [9]. Therefore, the United States Environmental Protection Agency (US EPA) and European Community (EC) regard DEHP as an environmental priority pollutant [10]. The reference dose (RfD), or tolerable daily intake (TDI), of DEHP is 20 mg/kg/d assessed by the US EPA (2007), and 37 mg/kg/d by the EU Scientific Committee for Toxicity, Ecotoxicity, and the Environment (CSTEE) [11]. So taking the omnipresence and toxicity of DEHP into consideration, the detoxication of such a contaminant seems especially important.

After entering the body, DEHP is first hydrolyzed into mono (2-Ethylhexyl) phthalate (MEHP) by biotransformation of phase I enzymes in the liver. Under the catalysis of UDP-glucuronosyltransferase (UGT), these monoesters and oxidation products were converted into glucuronic acid by phase II biotransformation, which enhanced their water solubility and reduced their biological activity; and they were then metabolized and excreted from urine [12,13,14,15]. Biotransformation of non-polar xenobiotics generally involves two systems, the phase I and phase II enzyme systems, with the most important system being the phase I system [16]. Key enzymes of the phase I system include the Cytochrome P450 (CYP450). Enzymes such as UGT and glutathione S-transferase (GST) play pivotal roles in the phase II conjugation reactions. Therefore, the quantity and/or activity of these key enzymes might be crucial in detoxication processes.

The pregnane X receptor (PXR), also known as the steroid and xenobiotic sensing nuclear receptor (SXR), is a protein that was discovered in 1998. The discovery of PXR provided a molecular basis for solving a long-standing problem in clinical medicine, of how specific drugs could induce pathways of drug clearance, and thereby enhance the rate of their own elimination, as well as co-administered drugs [17]. Pregnane X receptor (PXR) is the key to the body’s response to toxic exogenous substances and endogenous metabolites. By acting as a ligand-activated transcription factor, PXR regulates all stages of heterobiotic metabolism and transport, and is responsible for the important induction of drug interactions [17]. Activation of human PXR (hPXR) results in transcriptional induction of Cytochrome P450 3A4 (CYP3A4) phase II hepatic biotransformation enzyme genes, i.e., those encoding UGTs and sulfotransferases (SULTs) [18]. Following ligand-binding, PXR forms a complex with its obligate heterodimeric partner the retinoid X receptor alpha (RXRα) or nuclear receptor subfamily 2, group B, member 1 (NR2B1), and then binds to response elements in the promoters and enhancers of the target genes [19].

*Lycium barbarum* (LB), also known as Goji, is a popular ingredient in the preparation of traditional Chinese medicine [20]. LB is also known as wolfberry in the Western world, and has become studied for its health-promoting properties in recent years in Asia, Europe, and North America [21,22,23,24]. *Lycium barbarum* extract, especially LB polysaccharides (LBP) is a renowned antioxidant, and has been reported on for its antioxidant capacity in various in vitro [25], in vivo [26], and clinical studies [27]. Previous studies have shown that LBP can reduce the DNA damage of non-insulin dependent diabetes mellitus (NIDDM) rats by reducing the level of oxidative stress [26]; it also significantly inhibited the peroxidation loss of β - carotene in β - carotene linoleic acid system; scavenged DPPH free radicals [25]; significantly improved the activities of Superoxide dismutase (SOD) and Gluthathione peroxidase (GSH-Px) in adult serum; and reduced the content of MDA [27]. *Lycium barbarum* polysaccharide (LBP), also has functions in, increasing immunity [28], anticancer [29], anti-aging [30], and reproductive protection [31]. For example, LBP could significantly inhibit replicative senescence, and down regulate the senescence related genes [32]. In lipopolysaccharide (LPS) induced inflammation, LBP had a certain inhibitory effect on immune response [33]. *Lycium barbarum polysaccharide* could improve the retinal morphology and function of RD1 mice, and delay the functional decline of retinal ganglion cells in the process of photoreceptor degeneration [34].

Taking their antioxidative capacity into consideration, exploring the intervention effects of LBP and wolfberry juice (WJ) on DEHP toxicity might be promising. The present study aimed to establish an animal model of DEHP exposure, and use LBP and WJ as the active intervention substance, as well as to investigate the effects of LBP and WJ on PXR-mediated phase I and phase II metabolic enzyme activities following DEHP exposure. On this basis, we compared the effects of LBP and WJ on the repair of DEHP induced liver injury, and hope to provide a new method for improving liver injury.

## 2. Materials and Methods

### 2.1. Animals

A total of 120 male Sprague–Dawley (SD) rats, ranging in weight from 180–210 g, were purchased from Southern Medical University (Guangzhou, China). All experimental procedures were conducted in accordance with the NIH Guide for the Care and Use of Laboratory Animals. All animal tests were approved by the Animal Care Committee of the Laboratory Animal Center of South China Agricultural University, Guangzhou (#SCXK2011-0015). Animals were housed in stainless steel metabolic cages (one per cage) at constant room temperature (22 ± 0.5 °C) and relative humidity (50–60%), with a 12 h:12 h light–dark cycle. All rats had access to food and water ad libitum.

### 2.2. Design

#### 2.2.1. LBP Intervention before DEHP Exposure

After adaptation for 7 d, rats were divided into five groups (*n* = 8): control group (C), DEHP group (DEHP, 2000 mg DEHP/kg·bw), low-dose LBP intervention group (LBP-L, 2000 mg DEHP/kg·bw + 50 mg LBP/kg·bw), medium-dose LBP intervention group (LBP-M, 2000 mg DEHP/kg·bw + 100 mg LBP/kg·bw), and high-dose LBP intervention group (LBP-H, 2000 mg DEHP/kg·bw + 200 mg LBP/kg·bw). The LBP groups were given an indicated amount of LBP dissolved in saline by intragastric administration on a daily basis for 7 consecutive days, while the C group were treated with equal volumes of saline. At the 7th d after administration of LBP or saline, the rats were allowed to rest for one hour. After the rest, the DEHP and each of the LBP groups received a one-time gavage of 2000 mg DEHP/kg·bw dissolved in sesame oil, while the control group was treated with an equal volume of sesame oil. After a day off, the rats were sacrificed by a quick cervical dislocation. The serum was centrifuged after standing. Then, 1 g of liver was taken accurately, and washed with cold PBS. Then the liver was ground to prepare 10% liver homogenate, centrifuged (376 g, 20 min), and the supernatant was stored at −80 °C.

#### 2.2.2. LBP and WJ Intervention after DEHP Exposure

After adaptation for 7 d, the rats were divided into four groups (*n* = 8): control group (C), DEHP group (DEHP, 3000 mg DEHP/kg·bw), high-dose LBP intervention group (LBP-H, 3000 mg DEHP/kg·bw + 200 mg LBP/kg·bw), and wolfberry juice group (WJ, 3000 mg DEHP/kg·bw + 8 mL/kg·bw WJ). On the first day, the DEHP, LBP-H, and WJ groups received a one-time gavage of 3000 mg DEHP/kg·bw dissolved in sesame oil, while the control group was treated with an equal volume of sesame oil (in order to cause more obvious toxicity, to observe the detoxification effect of LBP-H and WJ, the concentration of DEHP was changed to 3000 mg/kg·bw). The gavage volume was converted according to 4 mL/kg. Waiting 1 h after DEHP administration, the LBP-H and WJ groups were given an indicated amount of LBP and wolfberry juice dissolved in saline by intragastric administration. On the next six days, the LBP-H and WJ groups were given an indicated amount of LBP and wolfberry juice dissolved in saline by intragastric administration on a daily basis, while the control group were treated with equal volumes of saline. On the 1st, 3rd, 5th, and 7th day after gavage, 5 rats were randomly sacrificed by a quick cervical dislocation. Refer to Section 2.2.1 for preparation of serum and liver homogenate samples.

### 2.3. Reagents

LBP was purchased from Shanghai Xian Pu Biological Technology Co. Ltd., Shanghai, China, Purity of the LBP was greater than 90%. WJ was purchased from Qinghai Dashu Red Medlar Co. Ltd., Qinghai, China.

### 2.4. ELISA Procedures

The hepatic activity of SOD, Glutathione (GSH), GSH-Px, Malondialdehyde (MDA), PXR, RXRα, CYP450, Cytochrome P450 2E1 (CYP2E1), Cytochrome P450 3A1 (CYP3A1), UGT1, GST, and Glutathione S-Transferase Pi (GSTpi) was analyzed using commercial ELISA kits, according to the manufacturers’ instructions (Laibio, Shanghai, China). A double antibody sandwich method was used. In the process of determination, the purified rat antibodies (SOD, GSH, GSH-Px, MDA, PXR, RXRα, CYP450, CYP2E1, CYP3A1, UGT1, GST, and GSTpi in the kit) reacted only with the corresponding antibodies in the samples. After adding enzyme labeled antigen or antibody, the substrate was catalyzed into colored products by enzyme. The content of the product was directly related to the content of the tested substance in the standard. Then, the concentrations of SOD, GSH, GSH-Px, MDA, PXR, RXRα, CYP450, CYP2E1, CYP3A1, UGT1, GST, and GSTpi were determined by comparing the outer diameter of the sample with the standard curve.

### 2.5. Statistical Analysis

All raw data were collated in a Microsoft Excel database, whilst SPSS 18.0 and Graphpad Prism were used to analyze data and make graphs, respectively. Experimental data were expressed as means ± standard deviation (means ± SD). Differences among groups were compared with one-way analysis of variance. The significant difference level was accepted at *p* < 0.05, and the extremely significant difference level was accepted at *p* < 0.01.

## 3. Results

### 3.1. Effects of LBP Intervention before DEHP Exposure on Serum and Liver Oxidative Stress Index in Rats

**Figure 1:**
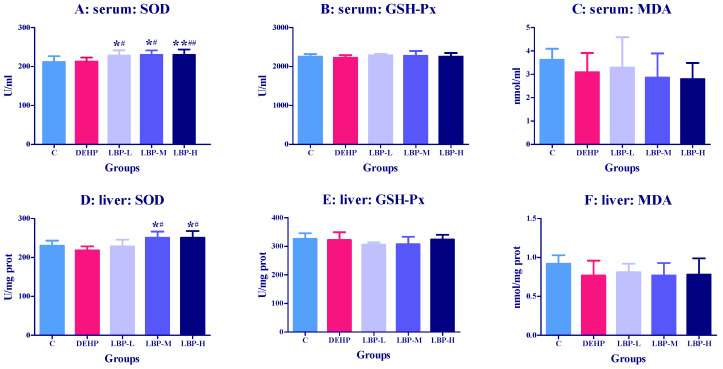
Effects of *Lycium barbarum* polysaccharide (LBP) intervention before Di(2-Ethylhexyl) phthalate (DEHP) exposure on serum and liver oxidative stress index in rats (*n* = 8). *Lycium barbarum* polysaccharide was continuously given for seven days, and DEHP was fed on the last day. The related oxidative stress indexes (SOD, SGH-Px, MDA) in serum and liver were determined by ELISA kit. (**A**,**D**): activities of SOD in serum and liver, respectively. (**B**,**E**): activities of GSH-Px in serum and liver, respectively. (**C**,**F**): concentrations of MDA in serum and liver, respectively. Data are expressed by mean ± S.D. The significance of difference among treatment groups was determined by one-way ANOVA. * or **: compared with control group, *p* < 0.05 or 0.01; ^#^ or ^##^: compared with DEHP group, *p* < 0.05 or 0.01.

As shown in Figure 1A, the activity of serum SOD in the LBP-L, LBP-M, and LBP-H groups was significantly increased (*p* < 0.05) compared with the control group and DEHP. The liver SOD activity of the LBP-M and LBP-H groups was higher than those of the control group and DEHP group (*p <* 0.05). With the intervention with LBP, the activity of SOD increased in a dose-dependent manner.

### 3.2. Effects of LBP Intervention before DEHP Exposure on Liver Metabolic Enzyme Activities in Rats

**Figure 2:**
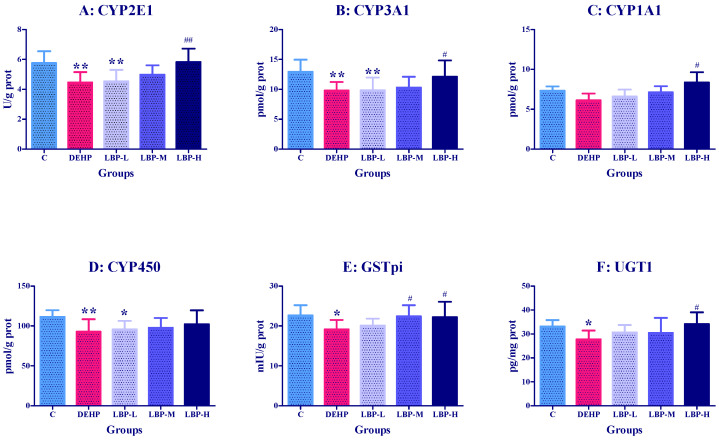
Effects of LBP intervention before DEHP exposure on liver metabolic enzyme activities in rats (*n* = 8). *Lycium barbarum* polysaccharide was continuously given for seven days, and DEHP was fed on the last day. The related liver metabolic enzyme activities (CYP2E1, CYP3A1, CYP1A1, CYP450, GSTpi, and UGT1) were determined by ELISA kit. (**A**–**F**): Concentrations or activities of CYP2E1, CYP3A1, CYP1A1, CYP450, GSTpi and UGT1 in liver, respectively. Data are expressed by mean ± S.D. The significance of differences among treatment groups was determined by one-way ANOVA. * or **: compared with control group, *p <* 0.05 or 0.01; ^#^ or ^##^: compared with DEHP group, *p <* 0.05 or 0.01.

The effects of LBP intervention on the liver phase I and phase II enzyme activities of CYP2E1, CYP3A1, CYP450, GST-pi, and UGT1 in the rats are shown in Figure 2. Compared with the control group, the activities or concentrations of CYP2E1, CYP3A1, CYP450, GSTpi, and UGT1 in the DEHP group were significantly decreased (*p* < 0.01 or *p <* 0.05). With the intervention with LBP, the activity of all phase I and phase II enzymes, except GSTpi, increased in a dose-dependent manner. The activities of CYP2E1, CYP3A1, and CYP450 were significantly lower than those in the control group. The activities of CYP2E1, CYP3A1, CYP1A1, and UGT1 in the LBP-H group were significantly higher than those in the DEHP group (*p <* 0.01 to *p <* 0.05), while the activity of GSTpi in the LBP-M and LBP-H groups was significantly higher than that in the DEHP group (*p <* 0.05). The activity of UGT1 in the LBP-H group was significantly higher than that in the DEHP group (*p <* 0.05).

### 3.3. Effects of LBP Intervention before DEHP Exposure on PXR Concentration in Rats

**Figure 3:**
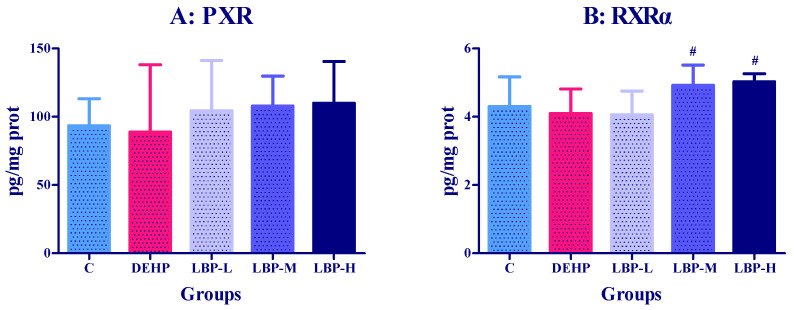
Effect of LBP intervention before DEHP exposure on transcriptional regulators in rats (*n* = 8). *Lycium barbarum* polysaccharide was continuously given for seven days, and DEHP was fed on the last day. The related liver metabolic enzyme activities (PXR and PXRα) were determined by ELISA kit. (**A**) or (**B**): concentrations of RXR or PXRα. Data are expressed by mean ± S.D. The significance of difference among treatment groups was determined by one-way ANOVA. #: compared with DEHP group, *p <* 0.05.

The effects of LBP intervention on PXR concentration in the rats are shown in Figure 3A. Pregnane X receptor concentration in the DEHP group was lower than that in the control group, but the difference was not significant. The PXR concentrations of LBP-L, M, and H group were higher than those of the DEHP group (*p* < 0.05). The PXR concentration increased with the intervention dose, but the difference was not significant. Similarly, the PXRα concentration of the DEHP group was lower than that of the control group, but the difference was not significant. The PXRα concentration increased with the increase of the intervention dose, and the concentrations of PXRα in the LBP-M and LBP-H groups were significantly higher than in the DEHP group (*p* < 0.05).

### 3.4. Effects of LBP and WJ Intervention after DEHP Exposure on Serum Oxidative Stress Index in Rats Exposed to DEHP

**Figure 4:**
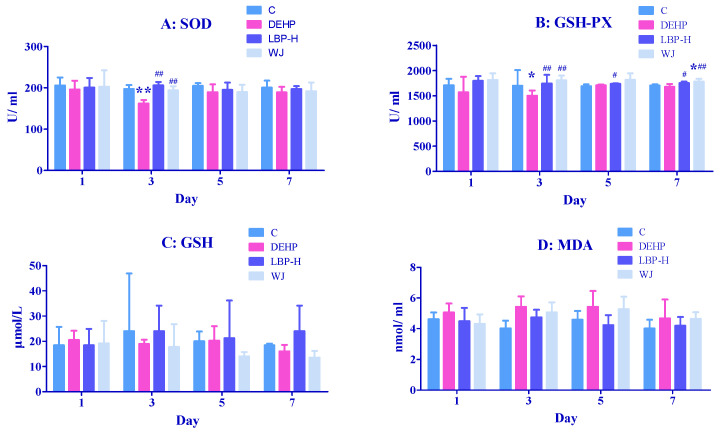
Effects of LBP and wolfberry juice (WJ) intervention on serum oxidative stress index in rats exposed to DEHP (*n* = 8). After one day of DEHP feeding, LBP and WJ were used for continuous intervention, and the serum oxidative stress indexes (SOD, SGH-Px, GSH, MDA) were measured by ELISA kit on the first, third, fifth, and seventh day of intervention. (**A**–**D**): concentrations and activities of SOD, GSH-Px, GSH, or MDA in serum, respectively. Note: data are expressed by mean ± S.D. The significance of difference among treatment groups was determined by one-way ANOVA. * or **: compared with control group, *p <* 0.05 or 0.01; ^#^ or ^##^: compared with DEHP group, *p <* 0.05 or 0.01.

The effects of LBP and WJ intervention on serum SOD, GSH-Px, GSH, and MDA in the rats exposed to DEHP are shown in Figure 4. On the third day, the SOD and GSH-PX activity in the DEHP group was lower compared with the control group (*p* < 0.05), and the activities of SOD and GSH-Px in the LBP-H and WJ groups were increased when compared with the DEHP group (*p* < 0.01). Compared with the DEHP group on the 5th and 7th day, the GSH-Px activity in the LBP-H group was significantly higher (*p* < 0.05). In addition, the WJ group augmented the levels of GSH-Px compared with the control group and DEHP group on the seventh day (*p* < 0.05).

### 3.5. Effects of LBP and WJ Intervention after DEHP Exposure on Liver Oxidative Stress Index in Rats Exposed to DEHP

**Figure 5:**
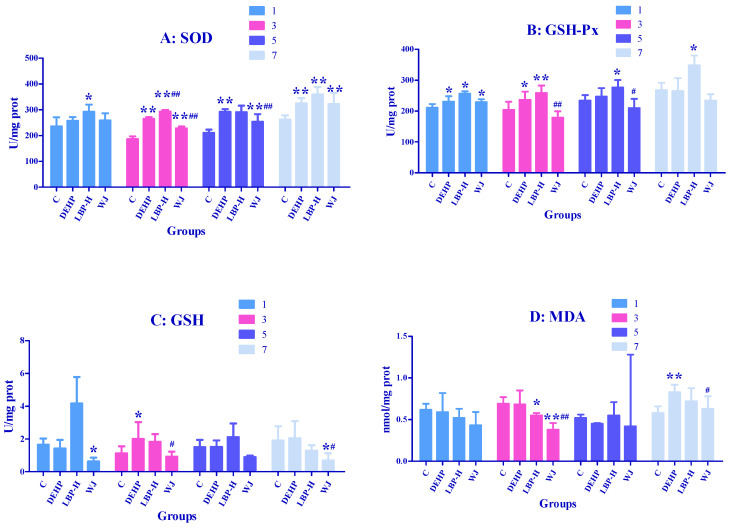
Effects of LBP and WJ intervention on liver oxidative stress index in rats exposed to DEHP (*n* = 5). After one day of DEHP feeding, LBP and WJ were used for continuous intervention, and the liver oxidative stress indexes (SOD, SGH-Px, GSH, MDA) were measured by ELISA kit on the first, third, fifth, and seventh day of intervention. (**A**–**D**): concentrations and activities of SOD, GSH-Px, GSH, and MDA in liver, respectively. Note: data are expressed by mean ± S.D. The significance of differences among treatment groups was determined by one-way ANOVA. * or **: compared with the control group, *p* < 0.05 or 0.01; ^#^ or ^##^: compared with DEHP group, *p* < 0.05 or 0.01.

As shown in Figure 5A, on the first day the activity of SOD in the LBP-H group was markedly higher than that of the control group (*p <* 0.05); On the third day, DEHP, LBP-H, and WJ had markedly elevated SOD activity compared with the control group (*p <* 0.01); the high-dose LBP intervention group (LBP-H) had significantly increased activity of SOD compared to the DEHP group (*p <* 0.01), while treatment with WJ had the opposite effect (*p <* 0.01). On fifth day the activity of SOD in the DEHP and WJ groups was significantly higher than that of control group (*p* < 0.01); wolfberry juice markedly inhibited the activity of SOD compared with the DEHP group (*p* < 0.01). On the seventh day DEHP, LBP-H, and WJ markedly elevated the activity of SOD compared with control group (*p* < 0.01). As shown in Figure 5B, on the first day the activities of GSH-Px in the DEHP, LBP-H, and WJ groups were significantly higher than that of the control group (*p* < 0.05). On the third day the DEHP and LBP-H groups had significantly increased GSH-Px activity compared with the control group (*p* < 0.05), while WJ significantly decreased GSH-Px activity compared with the DEHP group (*p* <0.01). On the fifth day, compared with the control group, the activity of GSH-PX in the LBP-H group was significantly higher (*p* < 0.05), while WJ significantly decreased GSH-Px activity compared with the DEHP group (*p* < 0.05). On the seventh day, LBP-H significantly increased the activity of GSH-Px compared with the DEHP group (*p* < 0.05). As shown in Figure 5C, on the first day WJ significantly decreased the activity of GSH compared with the control group (*p* < 0.05). On the third day the activity of GSH in the DEHP group was significantly higher than that of the control group (*p* < 0.05), and WJ significantly decreased GSH activity compared with the DEHP group (*p* < 0.05). On the seventh day the activity of GSH in the WJ group was significantly lower compared with the control and DEHP groups (*p* < 0.05). As shown in Figure 5D, on the third day LBP-H and WJ significantly decreased the level of MDA compared with the control group(*p* < 0.05), and the concentration of MDA was significantly lower than that of the DEHP group (*p* < 0.01). On the seventh day DEHP significantly increased the level of MDA compared with the control group (*p* < 0.01), while WJ significantly decreased the level of MDA compared with the DEHP group (*p* < 0.05).

### 3.6. Effects of LBP and WJ Intervention after DEHP Exposure on Liver Metabolic Enzyme Activities in Rats Exposed to DEHP

**Figure 6:**
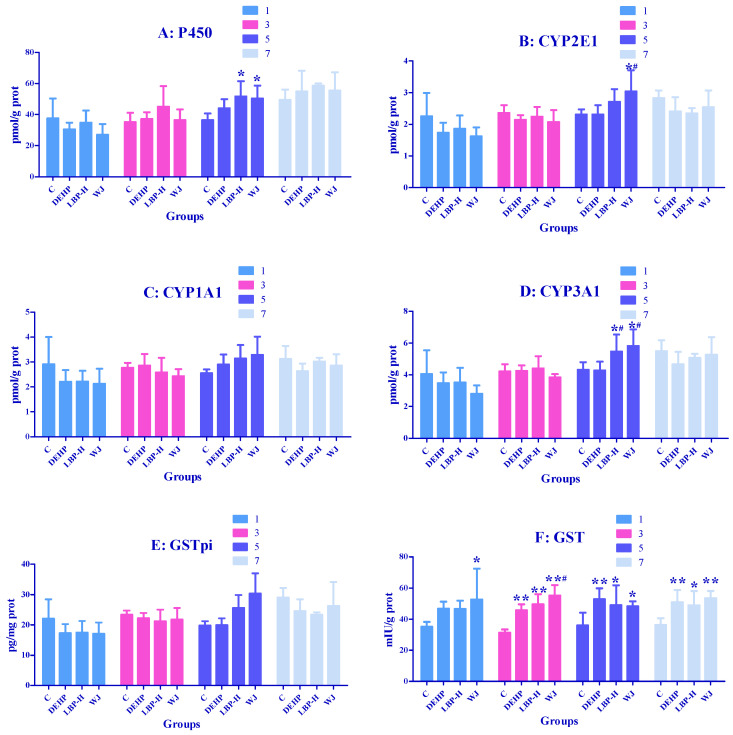
Effects of LBP and WJ intervention on liver metabolic enzyme exposed to DEHP activity in rats (*n* = 5). After one day of DEHP feeding, LBP and WJ were used for continuous intervention, and the liver metabolic enzymes (CYP450, CYP2E1, CYP1A1, CYP3A1, GSTpi, and GST) were measured by ELISA kit on the first, third, fifth, and seventh day of intervention. (**A**–**F**): concentrations and activities of CYP450, CYP2E1, CYP1A1, CYP3A1, GSTpi, and GST in liver, respectively. Note: data are expressed by mean ± S.D. The significance of differences among treatment groups was determined by one-way ANOVA. * or **: compared with control group, *p* < 0.05 or 0.01; ^#^ or ^##^: compared with DEHP group, *p* < 0.05 or 0.01.

As shown in Figure 6A, on the fifth day the levels of P450 in the LBP-H group and WJ group were markedly higher than those of the control group (*p* < 0.05). As shown in Figure 6B, on the fifth day compared with the control group and DEHP group, WJ markedly elevated the concentrations of CYP2E1 (*p* < 0.05); As shown in Figure 6D, on the fifth day LBP-H and WJ markedly increased the levels of CYP2E1 compared with the control group and DEHP group (*p* < 0.05). As shown in Figure 6F, on the first day WJ markedly increased the GST activity over that of the control group (*p* < 0.05). On the third day the activity of GST in the DEHP group, LBP-H group, and WJ group was markedly higher than that of the control group (*p* < 0.01), and WJ markedly increased the activity of GST compared with the DEHP group(*p* < 0.05). On fifth and seventh days the activity of GST in the DEHP group, LBP-H group, and WJ group was markedly higher compared to the control group (*p* < 0.05).

### 3.7. Effects of LBP and WJ Intervention after DEHP Exposure on PXR Activity in Rats Exposed to DEHP

**Figure 7:**
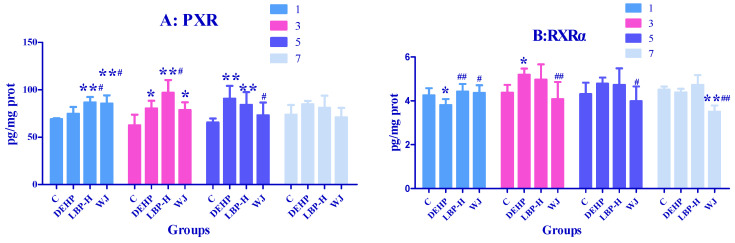
Effects of LBP and WJ intervention on transcriptional regulator activity in rats exposed to DEHP (*n* = 5). After one day of DEHP feeding, LBP and WJ were used for continuous intervention, and the transcriptional regulator activity (PXR and PXRα) was measured by ELISA kit on the first, third, fifth, and seventh day of intervention. This is the concentration of PXR(**A**) and PXRα(**B**), respectively. Note: data are expressed by mean ± S.D. The significance of differences among treatment groups was determined by one-way ANOVA. * or **: compared with control group, *p* < 0.05 or 0.01; ^#^ or ^##^: compared with DEHP group, *p* < 0.05 or 0.01.

As shown in Figure 7A, on the first day the levels of PXR in the LBP-H group and WJ group were significantly higher compared with the control group and DEHP group (*p* < 0.05). On the third day DEHP, LBP-H, and WJ significantly augmented PXR levels compared with the control group (*p* < 0.05), and LBP-H significantly increased the PXR concentration compared with the DEHP group (*p* < 0.05). On the fifth day the levels of PXR in the WJ group were significantly lower than those of the DEHP group (*p* < 0.05), and DEHP and LBP-H significantly increased PXR levels compared with the control group (*p* < 0.01). As shown in Figure 7B, on the first day DEHP significantly decreased the levels of PXRα compared with the control group (*p* < 0.05), while LBP-H and WJ significantly increased the levels of PXRα compared with the DEHP group (*p* < 0.05). On the third day DEHP increased PXRα concentrations compared to control (*p* < 0.05), and the level of WJ was significantly lower than that of the DEHP group (*p* < 0.01). On fifth day WJ significantly decreased PXRα levels compared with the DEHP group (*p* < 0.05). On the seventh day WJ significantly decreased PXRα concentrations compared with the control group and DEHP group (*p* < 0.01).

## 4. Discussion

### 4.1. LBP Intervention before DEHP Exposure

The analysis of oxidative damage in the rats before exposure showed that the SOD and GSH-Px activity of the serum and liver did not significantly alter, and the concentrations of MDA also did not change significantly, which indicated that DEHP did not cause obvious oxidative damage to the rats at the initial stage of exposure. However, LBP intervention can improve the SOD activity of the serum and liver, and enhance the antioxidant capacity of rats.

DEHP is a lipophilic compound with high absorption upon oral exposure. Cytochrome P450 determines the rate of exogenous clearance and the production of toxic (carcinogenic) products, whilst the CYP1, CYP2, and CYP3 families mediate the metabolism of the vast majority of xenobiotics in the human body. Among them, inducing the cytochrome P450 enzymatic system has been proposed to be one of the most sensitive biological responses for responding to environmental changes. A previous study showed that DEHP exposure for 7 d at a dose of 1700 mg/kg·bw, increased the activity of CYP450, CYP2E1, and CYP3A1, and reduced the activity of CYP1A1 in male rats [35,36]. Their use of DEHP dose was close to ours, and the results were similar. In the current study, the activity of CYP2E1, CYP3A1, and CYP450 in the DEHP group was significantly decreased, and no significant difference was found for CYP1A1. The discrepancies in results between the studies might be due to different exposure times and doses of DEHP. The activity of phase I metabolic enzymes gradually increased with an increase in exposure time. For example, Chen et al. treated HepG2 cells with DEHP for 24 h and 48 h, and the levels of CYP1A1 and CYP3A4 were different for the two time periods [37]. Glucuronic-acid binding is an important phase II metabolic pathway in the organism, mainly catalyzed by UGT. The liver is the major organ of glucuronidation. UGTs are mainly expressed in the liver, and a large number of glucuronidation reactions occur in this organ [38]. The cytosolic glutathione S-transferases (GSTs) protect cells against various xenobiotic, electrophilic compounds through conjugation with glutathione. This renders these toxins less harmful to the cells, and facilitates their excretion [39]. The relatively high concentrations of environmental pollutant residues found in rats are catalyzed by phase II detoxifying enzymes, GSTs and UGT, and metabolism mainly occurs in the liver. The catalyzed products are water-soluble metabolites, which are discharged with bile or urine to achieve detoxification [40]. Burghll et al. suggested that UGT could be divided into UGT1 and UGT2 families [41], which are involved in the metabolism of exogenous chemicals (such as phenol), and endogenous bilirubin metabolism and steroid metabolism. The UGT1 and UGT2 families can be divided into many subfamilies. Glutathione-S-transferases pi (GSTpi) is one of the GST isoenzymes. GSTpi is a phase II detoxifying enzyme that protects cells from injury caused by toxic chemicals and products of oxidative stress. In humans, polymorphisms of GSTpi affect substrate selectivity and stability, and increase susceptibility to the parkinsonism-inducing effects of environmental toxins [42]. Therefore, it is necessary to explore the toxic effects of DEHP on GSTpi and UGT1 in rat liver, and the detoxification effects of LBP on DEHP exposure. He et al. found that when SD rats were exposed to a single dose of 3000 mg/kg DEHP, the content of liver UGT1 decreased 24 h later [43]. Rataj et al. found that exposure to 4 ug/kg·bw 17β-estradiol (E2) for 3 d inhibited uterine UGT1 expression to 26% of the baseline in ovariectomized rats [44]. Different studies in vivo and in vitro have demonstrated that DEHP has a weak estrogenic activity. Eveillard et al. found that the expression of GSTpi decreased by 2.4-fold after exposure of 15-week-old mice to 1100 mg/kg·bw DEHP for 14 d [14]. In the present study using the intragastric administration of 2000 mg/kg DEHP, we found that the activities of UGT1 and GSTpi decreased, which is consistent with previous studies. After consulting the relevant literature on LBP intervention, it was found that the most used concentration was 100 mg/kg [45], so we set three doses of 50, 100, and 200 mg/kg on this basis. LBP high-dose intervention could significantly upregulate the activities of GSTpi and UGT1. These findings indicated that LBP intervention could antagonize the phase II enzyme damage induced by DEHP.

As a key transcription factor, PXR regulates the expression of the CYP3A4 gene and plays an important role in the metabolic transport and maintenance of the in vivo environment. PXR’s regulation of drug-metabolizing enzymes is very extensive, including phase I metabolic enzymes such as CYP3A4, and phase II metabolic enzymes such as UGT and GST [46]. Du et al. examined several phthalate monoesters for their ability to activate mouse and human PXR [47]. Male quail were exposed to different doses of DEHP by gavage treatment for 45 d, and the activity of PXR increased in a dose-dependent manner [48]. In the current study, PXR activity in the DEHP group was lower than that in the control group, but the difference was not significant. The reason why the above experimental results were different from those in the present study might be due to the differences in the total dose, and the duration of DEHP exposure. Our experiment was based on a one-time exposure, while the above exposure was for 45 d. PXRα is the most important retinoid X receptor. PXR could play a regulatory role only in the formation of dimers with PXRα, so the inhibition of PXR may be the reason for the decline in PXRα activity in rats. The activities of PXR and PXRα increased after LBP administration, resulting in the intervention effect on the toxicity induced by DEHP.

### 4.2. LBP and WJ Intervention after DEHP Exposure

Di(2-Ethylhexyl) phthalate exposure could induce oxidative stress in the liver in a short period of time. The activities of SOD, GSH, and GSH-Px increased abnormally, to resist the oxidative stress, and their activities increased with time. The increase of SOD may have been related to the compensatory increase of SOD activity caused by the accumulation of superoxide free radicals produced by the body itself. The increase of GSH-Px may have been due to the accumulation of hydrogen peroxide produced by the body itself, which lead to the compensatory increase of GSH-Px activity. This was consistent with the results of Wang’s study, that DEHP can increase the activities of SOD and GSH-Px, and the level of GSH in the liver of rats [49]. MDA levels remained in a normal range from day 1 to day 3, then decreased significantly on day 5, and increased significantly on day 7. It was suggested that the content of MDA decreased in the early stage of DEHP exposure due to the self-defense system of rats, while in the later stage, the level of MDA increased due to the failure of SOD and GSH to remove free radicals in time. It was found that the activities of SOD, GSH-Px, GSH, and MDA in rats exposed to DEHP could be reduced by WJ intervention, which indicated that WJ could reduce the oxidative stress caused by DEHP, and its active components could play an antioxidant role; surprisingly, LBP intervention further enhanced the activities of SOD and GSH-Px in rats, and the intervention effect was not as good as that of WJ. In this study, the LBP content in the intervention group was equivalent to 100 mg/kg·bw, so the intervention effect of LBP may have been related to its dosage [50]. In addition to LBP, *Lycium barbarum* contains antioxidant components such as ascorbic acid, riboflavin, carotene, thiamine, and nicotinic acid, so the LBP of WJ working together with other active ingredients may enhance the intervention effect.

After exposure to DEHP, the activity of GST in hepatocytes was increased. GST could catalyze the nucleophilic addition of reduced GSH with electrophilic organic compounds, which played an important role in the response to oxidative stress. In this study, the up regulation of GST activity may have been due to the compensatory increase caused by the accumulation of electrophilic organic compounds. The content of CYP2E1 and CYP3A1 in WJ was significantly increased on the fifth day after exposure, and the concentration of CYP3A1 was significantly increased by LBP and WJ on the fifth day. These results indicate that continuous intervention could upregulate the content of CYP2E1 and CYP3A1, and then levels returned to normal.

Di(2-Ethylhexyl) phthalate exposure can lead to an abnormal increase of liver metabolic enzymes. To this end, we measured the contents of nuclear receptors RXR and PXR α. Surprisingly, they also showed abnormal increase. PXRα was the most important retinol like X receptor. PXR could play a regulatory role only when it formed a dimer with PXRα, which may be the reason for the same change trend of PXR and PXRα activity after DEHP exposure; there have been similar results before. Di(2-Ethylhexyl) phthalate exposure triggered the aryl hydrocarbon receptor (AhR)/PXR/constitutive androstane receptor (CAR) pathway, and altered the transcription of cerebellar CYP enzymes isoforms in quails [47]. After LBP intervention, the PXR activity of the rats was significantly higher than that of the control group on the first and third days, and was higher than that of DEHP exposure group on the third day, which may have been due to the synergistic effect of DEHP and LBP. The changes of PXR and PXRα activity after WJ intervention were opposite to that of DEHP, indicating that WJ played a positive role in intervention and regulation.

## 5. Conclusions

In conclusion, feeding LBP before DEHP exposure, or LBP and WJ after DEHP exposure, improved the hepatotoxicity induced by DEHP, perhaps through increasing the activities of metabolic enzymes in phase I and II regulated by the PXR pathway; and the intervention effect of WJ was more significant. In the future, we will focus on the reasons why WJ works better, and the development of related products.

## Data Availability

Datas presented in this study are available on request from the Authors.
